# Evolution and Structural Diversification of PILS Putative Auxin Carriers in Plants

**DOI:** 10.3389/fpls.2012.00227

**Published:** 2012-10-12

**Authors:** Elena Feraru, Stanislav Vosolsobě, Mugurel I. Feraru, Jan Petrášek, Jürgen Kleine-Vehn

**Affiliations:** ^1^Department of Applied Genetics and Cell Biology, University of Natural Resources and Life SciencesVienna, Austria; ^2^Department of Faculty of Science, Experimental Plant Biology, Charles UniversityPrague, Czech Republic; ^3^Institute of Experimental Botany of the Academy of Sciences of the Czech RepublicPrague, Czech Republic

**Keywords:** PILS proteins, auxin, evolution, phylogeny, auxin metabolism, auxin homeostasis

## Abstract

The phytohormone auxin contributes to virtually every aspect of the plant development. The spatiotemporal distribution of auxin depends on a complex interplay between auxin metabolism and intercellular auxin transport. Intracellular auxin compartmentalization provides another link between auxin transport processes and auxin metabolism. The PIN-LIKES (PILS) putative auxin carriers localize to the endoplasmic reticulum (ER) and contribute to cellular auxin homeostasis. PILS proteins regulate intracellular auxin accumulation, the rate of auxin conjugation and, subsequently, affect nuclear auxin signaling. Here, we investigate sequence diversification of the PILS family in *Arabidopsis thaliana* and provide insights into the evolution of these novel putative auxin carriers in plants. Our data suggest that PILS proteins are conserved throughout the plant lineage and expanded during higher plant evolution. PILS proteins diversified early during plant evolution into three clades. Besides the ancient Clade I encompassing non-land plant species, PILS proteins evolved into two clades. The diversification of Clade II and Clade III occurred already at the level of non-vascular plant evolution and, hence, both clades contain vascular and non-vascular plant species. Nevertheless, Clade III contains fewer non- and increased numbers of vascular plants, indicating higher importance of Clade III for vascular plant evolution. Notably, PILS proteins are distinct and appear evolutionarily older than the prominent PIN-FORMED auxin carriers. Moreover, we revealed particular PILS sequence divergence in *Arabidopsis* and assume that these alterations could contribute to distinct gene regulations and protein functions.

## Introduction

Plant development is particularly flexible due to its postembryonic growth behavior, allowing individual adjustment of the body plan according to the environment (Finet and Jaillais, 2012). The phytohormone auxin is crucial for these adaptive responses and, hence, has drawn enormous research attention (Teale et al., 2008). The importance of auxin for plant development seems to be also reflected in the complex regulation of auxin perception and its spatiotemporal distribution (Vanneste and Friml, 2009). Up to date three auxin receptor classes have been suggested to jointly regulate auxin-signaling output. Most auxin responses have been assigned to the F-box proteins TRANSPORT INHIBITOR RESPONSE1/AUXIN SIGNALING F-BOX (TIR1/AFB). Auxin binding to the co-receptors TIR1/AFB and the AUXIN/INDOLE-3-ACETIC ACID (Aux/IAA) will initiate the proteasome-dependent degradation of the transcriptional repressors Aux/IAAs. The subsequent release of AUXIN RESPONSE FACTOR (ARF) transcription factors eventually leads to the transcriptional reprogramming of the respective cell (Leyser, 2006; Chapman and Estelle, 2009). Another F-box protein S-PHASE KINASE-ASSOCIATED PROTEIN 2A (SKP2A) also binds to auxin and might contribute to auxin-dependent modulation of the cell cycle (Jurado et al., 2010). Rapid and non-genomic auxin effects appear to be mainly perceived by the AUXIN BINDING PROTEIN1 (ABP1; Jones and Venis, 1989; Robert et al., 2010; Xu et al., 2010). However, ABP1 action might also affect auxin-dependent gene transcription and cell cycle regulation (Braun et al., 2008; Tromas et al., 2009).

Beside the complex cell type-dependent regulation of auxin signaling, also auxin metabolism is multifaceted. Several redundant auxin biosynthesis pathways determine auxin levels in various tissues and the decay/inactivation of auxin is regulated via oxidation or mostly reversible conjugation (Woodward and Bartel, 2005; Ruiz Rosquete et al., 2012; Zhao, 2012). Auxin metabolism is highly dynamic and has pronounced importance for the spatiotemporal regulation of auxin.

Intercellular (polar) auxin transport also determines cellular auxin levels (Zazímalová et al., 2010). The most prominent auxin carriers are AUXIN-RESISTANT1/LIKE AUX1 (AUX/LAX) influx carriers, ATP BINDING CASSETTE (ABC) auxin transporters of a MULTIDRUG RESISTANCE (MDR) subfamily, and the PIN-FORMED (PIN) auxin carriers (Bennett et al., 1996; Chen et al., 1998; Gälweiler et al., 1998; Luschnig et al., 1998; Müller et al., 1998; Utsuno et al., 1998; Geisler et al., 2005). PIN proteins have a particular developmental importance as their polar localization at a given cell side determines the direction of the intercellular auxin flow (Wisniewska et al., 2006). PIN proteins can be grouped into two subclasses according to the length of the central hydrophilic loop. Canonical PIN1-type auxin efflux carriers have a long loop, localize to the plasma membrane (PM) and perform a rate-limiting function in cellular auxin efflux (Petrásek et al., 2006). In contrast, PIN5 and PIN8 have a dramatically reduced central hydrophilic loop, localize to the endoplasmic reticulum (ER) and regulate intracellular auxin compartmentalization and homeostasis (Mravec et al., 2009; Bosco et al., 2012; Ding et al., 2012).

**Figure 1:**
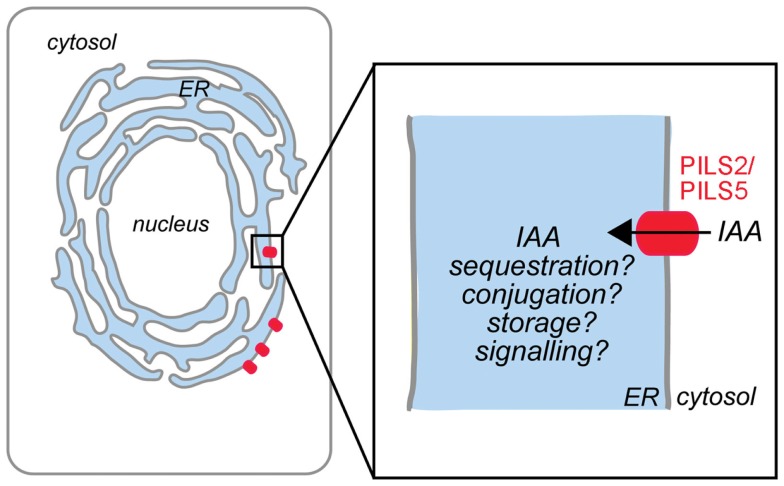
**Model of PILS protein function in *Arabidopsis thaliana***. PILS2 and PILS5 proteins localize to the ER and mediate intracellular auxin accumulation. We hypothesize that PILS proteins are putative auxin carriers that regulate the auxin transport from the cytosol into the lumen of the ER (black arrow). PILS activity affects auxin metabolism and might control the cytoplasmic availability of auxin (adapted from Barbez et al., 2012).

We have recently discovered a novel putative auxin carrier family of seven members in *Arabidopsis thaliana* (Barbez et al., 2012) and designated them as PIN-LIKES (PILS), because their predicted protein topology is highly similar to the topology of the PIN proteins. Similar to PIN proteins, PILS contain the so-called Interpro auxin carrier domain, an *in silico* defined domain to predict auxin transport function. Functional PILS5-GFP fusion proteins localize to the ER and stimulate intracellular auxin accumulation in plant and yeast cells (Barbez et al., 2012). PILS2 and PILS5 activity increases amide auxin conjugates, thereby reducing the free auxin levels, and negatively affecting nuclear auxin signaling (Barbez et al., 2012). Our current working model proposes that PILS2 and PILS5 proteins regulate auxin compartmentalization into the ER lumen, where auxin might be the substrate for compartmentalized auxin metabolism (Figure 1). It needs to be experimentally tested whether PILS proteins affect nuclear auxin signaling mainly by limiting the excess of auxin to diffuse into the nucleus or by the effect on presumably compartmentalized auxin conjugation. This mode of action is reminiscent to auxin carrier PIN5 that has been shown to regulate intracellular auxin homeostasis by modulating auxin compartmentalization and metabolism at the ER (Mravec et al., 2009). Further research will address whether the distinct protein families have redundant and interchangeable function at the ER.

PILS overexpression strongly distorts plant patterning and development, while *pils2* and *pils5* loss of function mutants show comparably weaker defects in plant growth regulation. Moderate PILS5 gain and *pils2pils5* loss of function phenotypes can be largely explained by low and high auxin content, respectively. For example, PILS5 overexpressors have reduced free auxin levels/signaling, shorter hypocotyls and fewer lateral roots, while *pils2pils5* double mutants display higher free auxin levels, enhanced hypocotyl growth and lateral rooting. In contrast, PILS5 gain and*pils2pils5* loss of function leads to reduced and enhanced root growth, which might be not related to the overall changes in auxin content, but could indicate a more specific PILS2 and PILS5 function in the cellular regulation of root growth (Barbez et al., 2012).

The identification of PILS proteins and their role in auxin homeostasis at the ER reveal the molecular complexity of intracellular auxin compartmentalization and its eminent importance for the plant development. Here we present *in silico* analysis to further reveal insight into the organization and regulation of this novel family of putative auxin transport facilitators.

## Material and Methods

### Sequence information

Sequences were downloaded from PLAZA^1^, NCBI^2^ by using tblastx program (Altschul et al., 1997; nr/nt database, PILS and PIN sequences from *A. thaliana* as queries) or Phytozome^3^ servers. The information and the ID of the presented sequences can be found in the Supplementary Data.

### Online servers

The online available servers used to perform *in silico* analyses of PILSes are found at: http://www.arabidopsis.org (chromosome localization; alternative splicing), http://bioinformatics.psb.ugent.be/plaza/ (intron/exon organization; sequence information), www.genevestigator.com (expression), http://bar.utoronto.ca/efp/cgi-bin/efpWeb.cgi (expression), http://www.enzim.hu/hmmtop/index.php (prediction of protein topology), http://biophysics.biol.uoa.gr/TMRPres2D (visual representation of transmembrane protein models), http://weblogo.berkeley.edu/logo.cgi (sequence logo), http://www.cbs.dtu.dk/services/NetPhos/ (prediction of phosphorylation sites), http://www.cbs.dtu.dk/services/NetPhosK (prediction of phosphorylation sites), http://www.ebi.ac.uk/Tools/msa/clustalw2/ (multiple amino acid sequences alignment), http://blast.ncbi.nlm.nih.gov/ (sequence information), http://www.phytozome.net/ (sequence information), http://www.r-project.org/ (analysis of collinearity).

### Analysis of collinearity

We investigated possible collinearity among *A. thaliana PILS* genes by comparing 200 surrounding translated genes for each *PILS*. The comparison was performed for pairs of *PILS* genes by using blastp program (Altschul et al., 1997). The homology was determined according to *E-value* from blast results. The analysis was performed in R environment^4^.

### Phylogenetic analysis

A multiple alignment was built by using Muscle in MEGA5 software (Tamura et al., 2011). Only the conserved domains were used and all positions with less than 80% site coverage were eliminated. The evolutionary history was inferred by using the Maximum Likelihood method based on the Whelan and Goldman (2001) + Freq. model with discrete Gamma distribution (five categories, *G* parameter = 3.0640) for analysis of PILS amino acid sequences or on the Whelan and Goldman model with discrete Gamma distribution (five categories, *G* parameter = 2.9920) for analysis of PIN-PILS dataset. The trees are drawn to scale, with branch lengths measured in the number of substitutions per site. The PILS analysis involved 42 amino acid sequences and 322 positions in the final dataset. The PIN-PILS analysis involved 67 amino acid sequences and 354 positions. Evolutionary analyses were conducted in MEGA5 (Tamura et al., 2011). For the sequence alignments see Figures S2 and S3 in Supplementary Material.

## Results

### Phylogeny of PILS proteins

Using available online tools, we previously showed that PILS proteins are highly conserved among plant species (Barbez et al., 2012). To further investigate the evolution of PILS protein diversification, we analyzed PILS protein sequences from all sequenced taxa of Viridiplantae. The PILS family is present in all the 26 available sequenced genomes and is represented by 202 genes (Table 1; Van Bel et al., 2012; confirmed by reciprocal blast, Altschul et al., 1997). PILS family obviously diversified in the different plant lineages (Table 1). Ancient species, such as algae (1–2), mosses (5), and spike mosses (8), have 1–8 *PILS* genes, while seed plants, such as *Oryza* (6), *Zea* (10), *Medicago* (13), or *Populus* (18), have 6 to 18 *PILS* genes (Table 1). The steadily increasing number in seed plants suggests that *PILS* genes have duplicated independently in several plant lineages and indicate a more diversified function of PILS proteins in higher plants.

**Table 1:** **Size of *PILS* gene families in different plant species**.

**Lineage**	**Species**	Number of genes*
**Eukaryota**		
Viridiplantae		
Chlorophyta	*Micromonas* sp.	1
	*Micromonas pusilla*	1
	*Ostreococcus lucimarinus*	1
	*Ostreococcus tauri*	1
	*Chlamydomonas reinhardtii*	2
	*Volvox carteri*	2
Bryophyta	*Physcomitrella patens*	5
Lycopodiophyta	*Selaginella moellendorffii*	8
Euphyllophyta		
Monocots	*Oryza sativa japonica*	6
	*Oryza sativa indica*	6
	*Sorghum bicolor*	7
	*Brachypodium distachyon*	8
	*Zea mays*	10
Dicots	*Carica papaya*	4
	*Arabidopsis lyrata*	6
	*Arabidopsis thaliana*	7
	*Ricinus communis*	7
	*Fragaria vesca*	8
	*Manihot esculenta*	9
	*Lotus japonicus*	12
	*Medicago truncatula*	13
	*Vitis vinifera*	13
	*Malus domestica*	14
	*Theobroma cacao*	16
	*Glycine max*	17
	*Populus trichocarpa*	18

*Gene information and sequences were retrieved from PLAZA platform (Van Bel et al., 2012) and candidates were evaluated by reciprocal blasts (Altschul et al., 1997).

**Figure 2:**
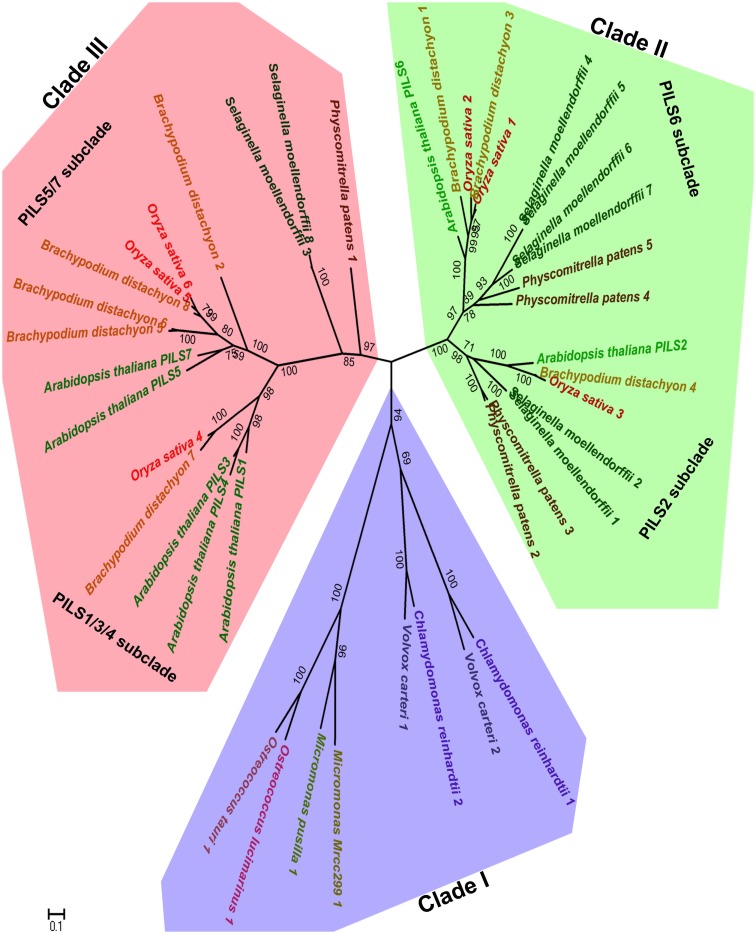
**Phylogeny of PILS proteins**. The phylogenetic tree of PILS proteins can be divided into three clades: Clade I (blue), Clade II (green), and Clade III (red). The *Arabidopsis* PILSes are found in the Clade II and Clade III, while Clade I is represented only by PILS proteins from algae. The Maximum Likelihood molecular phylogenetic analysis was performed in MEGA5 (Tamura et al., 2011) by using 42 amino acid PILS sequences from algae, *Physcomitrella*, *Selaginella*, *Brachypodium*, *Oryza*, and *Arabidopsis* as explained in the Materials and Methods.

To assess the evolutionary relationship among PILS proteins, we constructed phylogenetic trees with PILS sequences from selected model organisms such as available green algae, *Physcomitrella*, *Selaginella*, *Picea*, *Brachypodium*, *Oryza*, *Medicago*, *Arabidopsis*, and *Populus* sequences (Figure 2; Figure S1 in Supplementary Material; for sequence alignment see Figure S2 in Supplementary Material). The phylogenetic tree presented in Figure 2 shows that PILSes from Viridiplantae can be grouped into three evolutionary clades: Clade I, Clade II, and Clade III.

The available green algae genomes from the lineage *Chlorophyta* have a relatively low number of only one or two *PILS* genes per species. All these PILS algae orthologs cluster together and define the Clade I that contains the so far oldest known *PILS* genes of the Viridiplantae (Figure 2). We could also identify putative *PILS* genes in the genomes of sequenced algae from lineage Streptophyta from which the land plants evolved. However, we did not include these sequences in the phylogenetic analysis because they were incomplete (only EST fragments are currently available).

The evolutionary Clade II and III already emerged early during non-vascular plant evolution and both contain PILS sequences from Embryophytes (land plants; Figure 2). The main lineages of land plants are mosses, liverworts, hornworts, lycophytes, ferns, gymnosperms, and angiosperms. Clade II includes the well-conserved PILS2- and PILS6-like subclades, including orthologs of PILS2 and PILS6 from *Physcomitrella*, *Selaginella*, *Brachypodium*, or *Oryza* (Figure 2).

Clade III encompasses the PILS1/PILS3/PILS4- and PILS5/PILS7-like subclades and displays particular expansion in higher seed plants (Figure 2; Figure S1 in Supplementary Material). Accordingly, this clade encompasses also most *Brachypodium* and *Oryza* orthologs (Figure 2; Figure S1 in Supplementary Material). Interestingly, one *Physcomitrella* and two *Selaginella* PILS sequences are present at the root of the Clade III (Figure 2). The relatively low number of moss and the relative over amount of higher plant sequences in Clade III may suggest particular importance of this clade in vascular plant evolution.

Our analysis reveals that PILS proteins are evolutionarily conserved throughout plant evolution and might uncover the versatile importance of compartmentalized auxin homeostasis throughout the plant kingdom.

### PILS proteins are evolutionarily distinct of PIN proteins

The canonical PIN proteins act in the cellular efflux of auxin at the plasma membrane, but the most ancient members of PIN proteins (PIN5-type) localize to the ER and regulate the subcellular compartmentalization of auxin and auxin metabolism (Mravec et al., 2009). Hence, both PILS and PIN5-like proteins localize to the ER and regulate auxin homeostasis, presumably by mediating auxin transport at the ER (Mravec et al., 2009; Barbez et al., 2012; Bosco et al., 2012; Ding et al., 2012).

**Figure 3:**
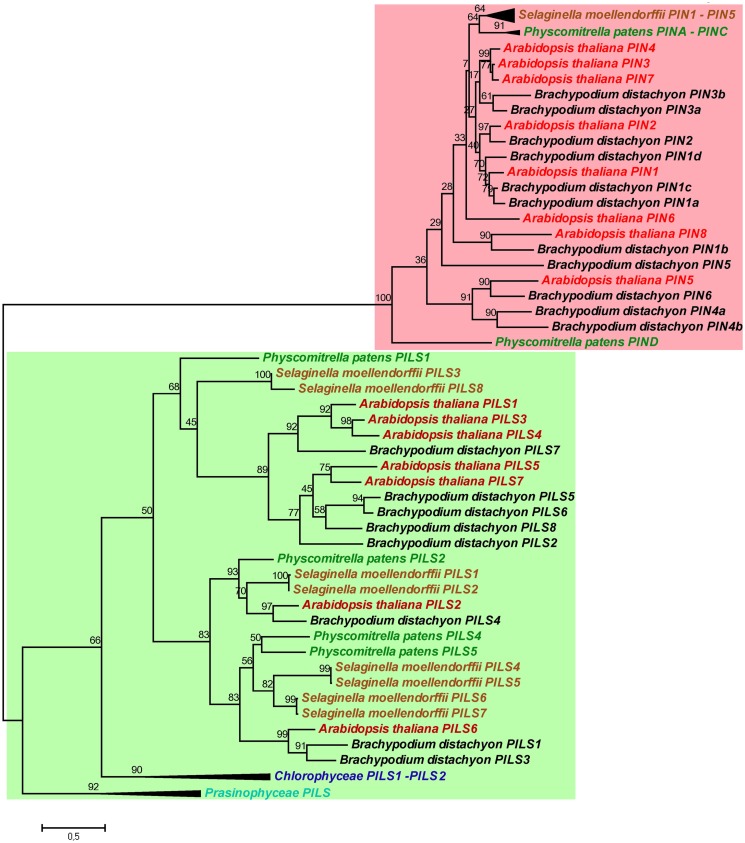
**Phylogeny of PILS and PIN proteins**. PILS proteins are evolutionarily distinct of PIN proteins. Note the two separated subtrees. The Maximum Likelihood phylogenetic analysis was performed in MEGA5 (Tamura et al., 2011) by using PILS and PIN amino acid sequences from algae, *Physcomitrella*, *Selaginella*, *Brachypodium*, and *Arabidopsis*. For a better visualization the algae and sometimes the *Physcomitrella* and *Selaginella* branches were collapsed.

Next, we investigated the evolutionary relationship between PILS and PIN proteins (Figure 3; for sequence alignment see Figure S3 in Supplementary Material). The phylogenetic analysis of PILS and PIN sequences from algae, moss, spikemoss, and several Angiosperms revealed that PILSes and PINs form two distinct phylogenetic clades (Figure 3). Although having a similar predicted protein structure and possibly similar function at the ER, PIN and PILS proteins are evolutionarily distinct in plants. In contrast to PILSes, we could not find any PIN sequence in the genomes of *Chlorophyta* algae, such as *Chlamydomonas*, *Micromonas*, *Ostreococcus*, *or Volvox*. Notably, a truncated PIN sequence has been found in the genome of *Spirogyra* (De Smet et al., 2011). These findings indicate that PILS proteins are more conserved during plant evolution and seem evolutionarily older than PIN proteins. Therefore, we assume that the PILS proteins are central to the evolution of intracellular auxin transport, which presumably has preceded the evolution of PIN-dependent intercellular and intracellular auxin transport.

### *PILS* diversification in *Arabidopsis thaliana*

**Figure 4:**
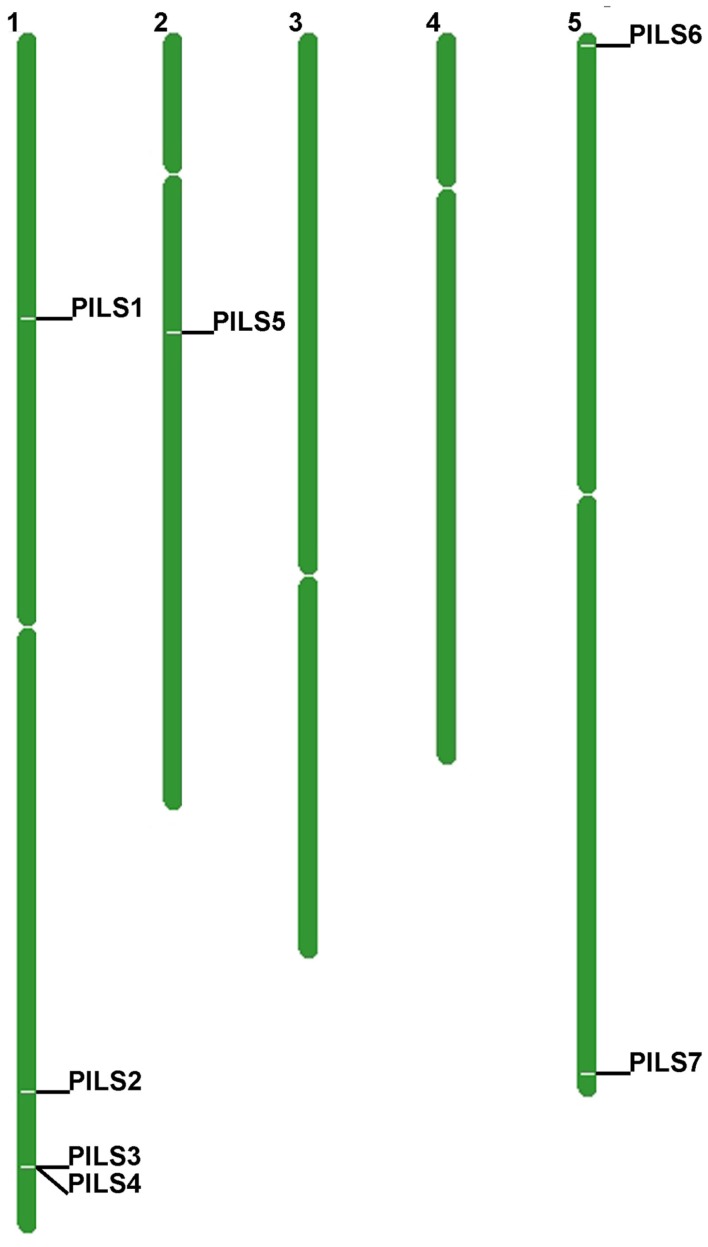
**Chromosomal distribution of *Arabidopsis thaliana PILS* genes**. *A. thaliana PILS* genes are found on chromosome 1, 2, and 5.

**Figure 5:**
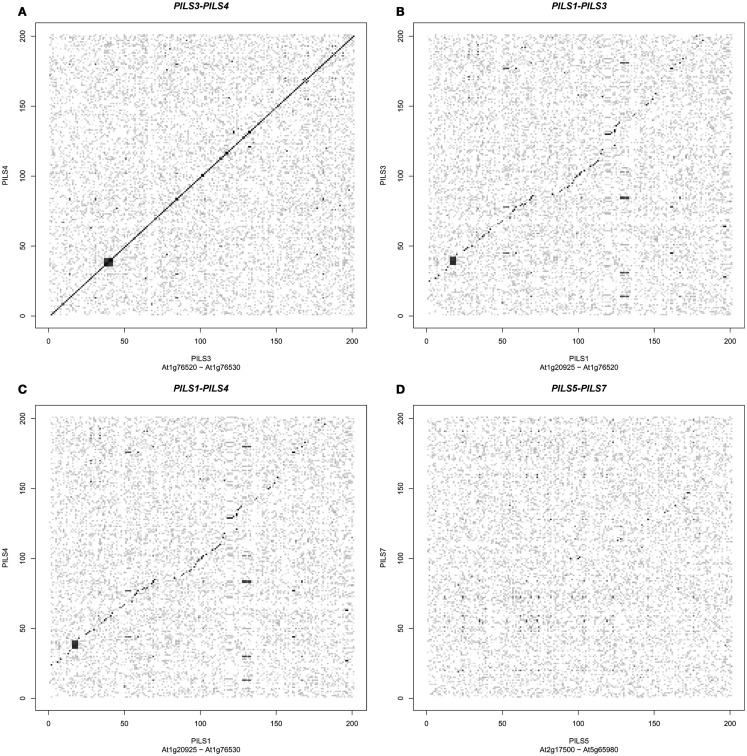
**Origin of *PILS* paralogs in *Arabidopsis thaliana***. **(A–D)** *PILS* genes show high collinearity between gene pairs *PILS3*/*PILS4* **(A)**, *PILS1*/*PILS3* **(B)**, and *PILS1*/*PILS4* **(C)**. No or only very weak collinearity could be detected between *PILS5* and *PILS7* **(D)**.

The seven *Arabidopsis PILS* genes are placed on chromosome 1, 2, and 5 (Figure 4). *PILS1* to *PILS4* are found on chromosome 1, *PILS5* on chromosome 2, while *PILS6* and *PILS7* are both placed at the ends of the chromosome 5 (Figure 4). *PILS3* and *PILS4* are neighboring genes at the bottom arm of the chromosome 1 (Figure 4), indicating that *PILS3* and *PILS4* may resulted from a gene duplication event. To investigate *PILS* paralogs in *A. thaliana* we performed comparative sequence analysis of genes that surround the seven *PILS* genes (Figure 5). Rows of 200 translated genes surrounding each of the seven *PILS* genes were analyzed in pairs by blastp program (Altschul et al., 1997) and homology between all genes in all unique pairs of gene rows were determined according to *E-value* from blast results. Pairs of gene rows with high diagonal homology were assigned as collinearity. In the *PILS1/PILS3/PILS4* group we found very high collinearity between *PILS3* and *PILS4* (Figure 5A). These genes appear to be products of very recent gene duplication. Between *PILS1* and the *PILS3/PILS4* pair we also found high collinearity (Figures 5B,C) and assume that these genes arose during full-genome duplication at Brassicaceae family level (20 million years ago; *Mya*). Only very weak or no collinearity was detectable between *PILS5* and *PILS7* (Figure 5D).

To further elaborate on the recent duplication of *PILS3* and *PILS4*, we analyzed the microevolutionary relationship between PILS sequences of *A. thaliana* and *A. lyrata* (Figure S1 in Supplementary Material). *A. lyrata* is the closest known relative of *A. thaliana* and has a genome of eight chromosomes and six PILS proteins (Van Bel et al., 2012). In contrast, *A. thaliana* has five chromosomes and seven PILS proteins (Barbez et al., 2012; Van Bel et al., 2012). It has been shown that the reduction of genome size in *A. thaliana* is the result of chromosomes fusion that presumably occurred about 5 *Mya* (Yogeeswaran et al., 2005). The phylogenetic analysis revealed that all six *A. lyrata* PILSes have highly similar orthologs in *A. thaliana*, while *AtPILS4* is a lineage-specific gene (Figure S1 in Supplementary Material). This indicates that *AtPILS4* is a duplicated gene that has arisen after the separation of *A. thaliana* from *A. lyrata* 5 *Mya*.

Next we addressed the sequence diversifications among the *A. thaliana* PILS proteins and performed a ClustalW Multiple sequence alignment (Larkin et al., 2007; Table 2; for sequence alignment see Figure S4 in Supplementary Material). PILS3/PILS4 showed the highest identity (82%), followed by PILS1/PILS3 (69%), PILS5/PILS7 (64%), and PILS1/PILS4 (61%; Table 2). Interestingly, PILS2 and PILS6 showed sequence identity of only 39% (Table 2). Taking together, the amino acid identity proposes that *A. thaliana* PILS proteins belong to three subgroups: (i) PILS1/PILS3/PILS4, (ii) PILS5/PILS7, and (iii) PILS2/PIL6.

**Table 2:** Percentages* of *Arabidopsis thaliana* PILS amino acid sequence identity calculated by ClustalW multiple sequence alignment (Larkin et al., 2007).

							PILS7
						31	PILS6
					31	64	PILS5
				39	28	43	PILS4
			82	41	31	43	PILS3
		32	28	29	39	30	PILS2
	28	69	61	40	29	42	PILS1
PILS1	PILS2	PILS3	PILS4	PILS5	PILS6	PILS7	

*The identity percentages were calculated as the identities between two PILS sequences, divided by the length of the alignment.

### *PILS* gene regulation and organization in *Arabidopsis thaliana*

**Figure 6:**
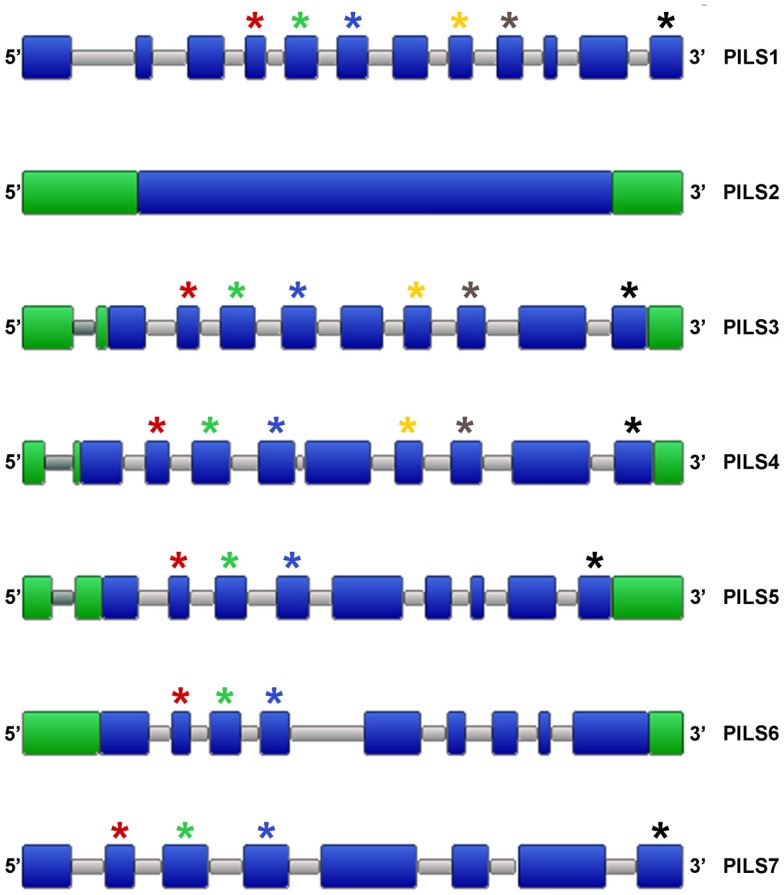
**Organization of *Arabidopsis thaliana PILS* genes**. Schematic intron/exon representation of *A. thaliana PILS* genes (Van Bel et al., 2012). Exons and introns are depicted in blue and gray, respectively. 3′ UTR and 5′ UTR are in green. Stars are showing exons of similar sizes (nucleotides): red (80), green (125), blue (122), yellow (93), gray (101), and black (125).

To get further insight into the regulation of PILS activity, we analyzed *in silico PILS* gene organization and expression. *A. thaliana PILS* gene transcripts organization is pretty well-conserved regarding the number and size of the exons (Figure 6). *PILS3* to *PILS6* genes contain nine exons with more or less conserved size and placements (Figure 6). In contrast, *PILS1*, *PILS2*, and *PILS7* have a divergent exon/intron structure. *PILS1* has 12 exons, *PILS7* bares eight exons and *PILS2* is even intron less. The size of exon number 2 (80 nucleotides), 3 (125 nucleotides), and 4 (122 nucleotides) is largely kept in *AtPILS* genes and encode for a highly conserved region of the predicted transmembrane helices 2–4 (109 aa in total). Also a C-terminal transmembrane domain seems to be encoded by the last exon (125 nucleotides) in almost all *AtPILS* genes (Figure 6).

**Figure 7:**
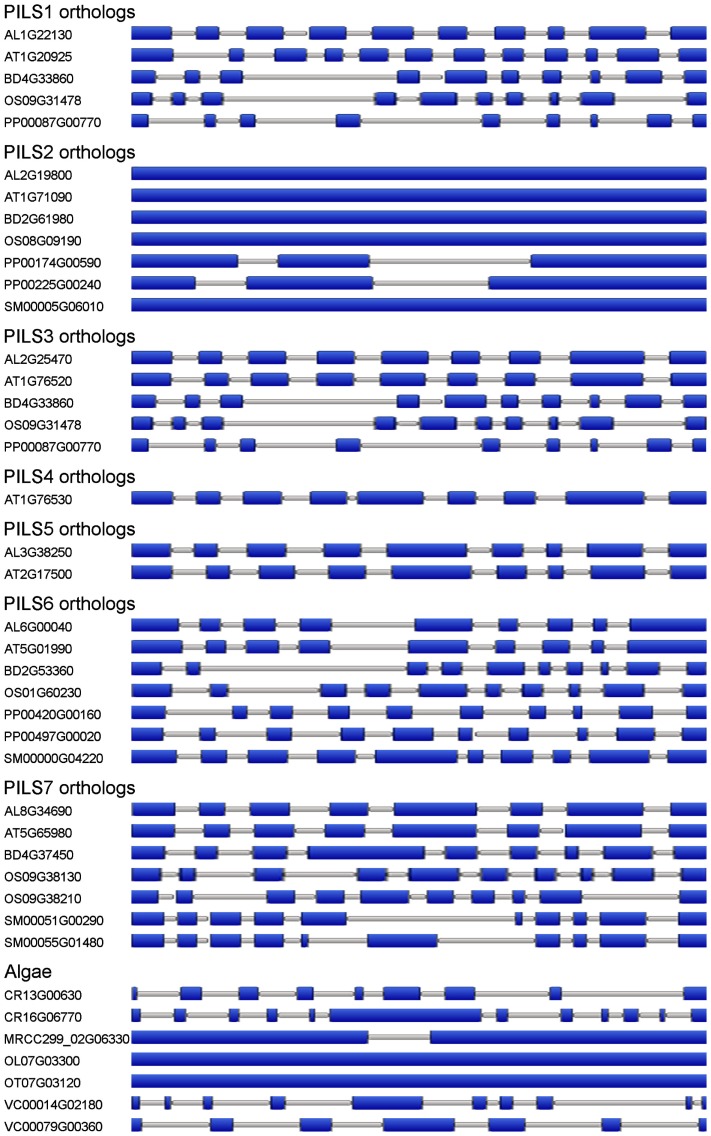
**Organization of *PILS* orthologs**. Schematic intron/exon representation of *PILS* genes from *Arabidopsis lyrata* (AL), *Arabidopsis thaliana* (AT), *Brachypodium distachyon* (BD), *Chlamydomonas reinhardtii* (CR), *Micromonas* (MRCC299), *Oryza sativa* (OS), *Ostreococcus lucimarinus* (OL), *Ostreococcus tauri* (OT), *Physcomitrella patens* (PP), *Selaginella moellendorffii* (SM), and *Volvox carteri* (VC; Van Bel et al., 2012). Exons are depicted in blue boxes, introns in gray lines.

Next, we analyzed the intron/exon organization of *PILS* genes from algae, *Physcomitrella*, *Selaginella*, and several Angiosperms. Our results show that *PILS* intron/exon organization is largely conserved among *PILS* orthologous (Figure 7). The variations of 1–2 more or less exons may be the result of insertions, deletions, or both processes along the lineage evolution. The subfamily of *PILS2* genes is most particular, because they display single-exon genes in Angiosperms and *Selaginella* and 3-exons genes in *Physcomitrella* (Figure 7). Thus, *PILS* genes belong to two structural groups with 1–3 exons (*PILS2* orthologs and *PILS* genes from *Ostreococcus* and *Micromonas*) and 7–12 exons (all the other *PILSes*; Figure 7).

**Figure 8:**
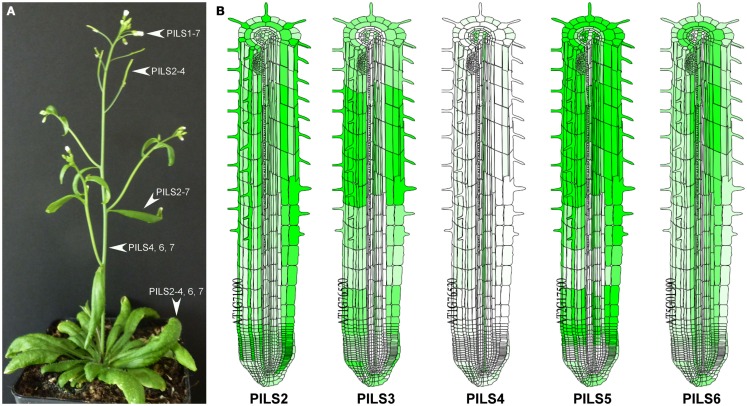
**Transcription pattern of *Arabidopsis thaliana PILS* genes**. **(A,B)** Transcription of *PILS* genes in *A. thaliana* plant **(A)** and root **(B)**. Based on the RT-PCR data from individual organs published in Barbez et al. (2012). **(A)** As predicted by online server *Arabidopsis* eFP Browser (http://bar.utoronto.ca/efp/cgi-bin/efpWeb.cgi); **(B)**.

*PILS* gene activity can be detected in all tissues of *A. thaliana* as shown by RT-PCR (Barbez et al., 2012) or by micro array-based online tools such as Genevestigator^5^. *PILS* genes display either relatively low (*PILS1*, *PILS4*, *PILS7*), medium (*PILS6*) or high (*PILS2*, *PILS3*, and *PILS5*) expression levels (see text footnote 5). *PILS2*-to-*AtPILS6* are expressed in seedlings, leaves, and flowers (Figure 8; Barbez et al., 2012; see text footnote 5). *PILS4* displays the strongest expression in the rosette leaves (Barbez et al., 2012). *PILS6* transcripts are particularly abundant in the stem and together with *PILS5* in the cauline leaves and flowers, while *PILS2* is highest in siliques (Barbez et al., 2012). Interestingly, some *PILS* gene products were excluded from certain tissues. *PILS1* was found to be expressed only in flowers, *PILS2* and *PILS3* are not expressed in the stem, *PILS5* is absent in the rosette leaves, stem, and siliques, while *PILS6* and *PILS7* were present in all plant organs but not in siliques (Barbez et al., 2012). Except *PILS1*, all the other *PILS*es were expressed in seedlings, with *PILS5* and *PILS2* having the highest expression (Barbez et al., 2012). *PILS2*-to-*PILS6* showed expression in pollen with *PILS5* being the most abundant (see text footnote 5). Based on these evidences it seems that *PILS* genes show specific and partially overlapping expression patterns in all plant tissues.

Alternative splicing might furthermore contribute to the regulatory complexity and diversity for *PILS* gene activity. *PILS3* and *PILS5* appear to bear two and four alternative transcripts, respectively^6^. In both cases the alternative gene splicing seems to occur in the 5′ region and may modulate *PILS3* and *PILS5* function. However, the importance of *PILS* transcript splicing remains to be demonstrated.

The pronounced differences in the expression levels and tissue distributions might indicate that PILS-mediated regulation of plant growth and development may be largely determined by gene regulation.

### PILS protein organization in *Arabidopsis thaliana*

**Figure 9:**
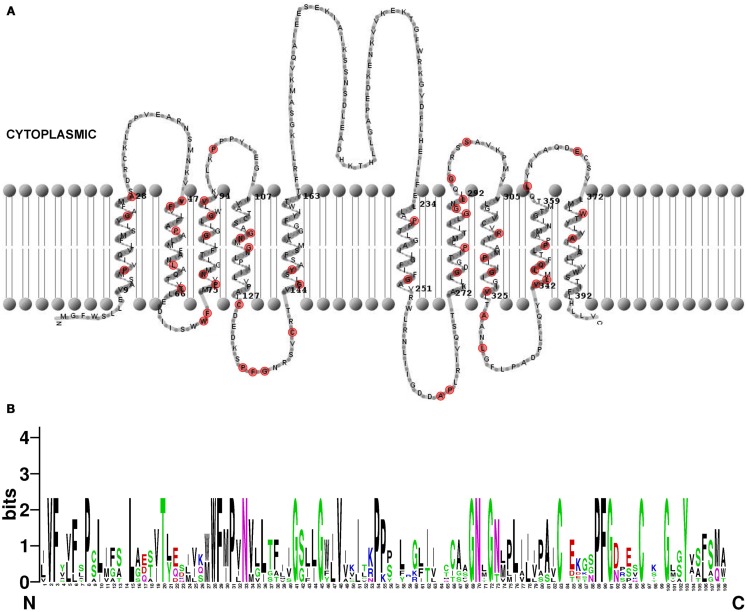
**Structure of *Arabidopsis thaliana* PILS proteins**. **(A)** Predicted topology of *A. thaliana* PILS5 protein. The prediction was done by HMMTOP 2.0 (Tusnády and Simon, 1998, 2001) and visualized by TMRPres2D (Spyropoulos et al., 2004). Conserved amino acids in all seven PILS proteins are marked in red. **(B)** Sequence logos generated by WebLogo (Schneider and Stephens, 1990) representing a ClustalW multiple sequence alignment (Larkin et al., 2007) of 109 amino acids from N-terminal region of *A. thaliana* PILS proteins (exons 2–4). Note the PILS sequence conservation at the highest, single symbol positions.

The temporal and spatial regulation of *PILS* genes will give rise to tissue specific distribution of distinct PILS proteins. Next we analyzed predicted PILS protein organization and searched for domains to speculate on PILS function. PILS proteins range in size from 390 (43 kDa; PILS3) to 472 (52 kDa; PILS1) amino acids. However, the predicted protein topology is highly similar for all PILS proteins. PILS proteins are presumably characterized by two hydrophobic transmembrane regions found at N- and C-termini (Figure 9A; Tusnády and Simon, 1998, 2001; Spyropoulos et al., 2004). The two transmembrane regions flank a short hydrophilic region (loop) with a presumable cytosolic orientation (Figure 9A). Each hydrophobic region appears to be organized in five transmembrane helices that are very similar and highly conserved among the PILS proteins (Figure 9). In contrast, the loop is less conserved and is the most divergent part of the PILS sequences. We assume that the transmembrane domains have central roles in the putative carrier function, while the presumably cytosolic loop might have rather regulatory functions.

PILS and PIN proteins share only 10–18% sequence identity and belong to distinct protein families (Figure 3; Barbez et al., 2012). However, the predicted topology of PILS proteins is reminiscent to the predicted topology of PIN proteins (Krecek et al., 2009) and allowed the identification of this novel putative auxin carrier family (Barbez et al., 2012). Based on the hydrophilic loop size, PIN proteins are sub-grouped into two subfamilies. The subfamily of PIN1-type encompasses the PIN members with a long hydrophilic loop and PM localization (PIN1–PIN4, PIN7), while the subfamily of PIN5-type encompasses PIN5 and PIN8 that have very short hydrophilic loops and ER localization. Although PIN6 shows a reduction of the loop size, PIN6 is often included in the PIN1-type subfamily due to high sequence similarity in the transmembrane regions (Krecek et al., 2009). However, it is also localized to the ER in transient localization studies (Mravec et al., 2009).

Similarly to PIN proteins, PILS family members are characterized by the presence of the Interpro auxin carrier domain. This Interpro domain is relatively long and spans almost the whole length of the PILS protein and, hence, it is difficult to ascertain functional residues within the “domain”.

Nothing is yet known about the post-translational modification of PILS proteins but generic phosphorylation sites (non-kinase-specific, such as serine, threonine, and tyrosine), kinase-specific phosphorylation sites and isoform variations could be predicted for PILS proteins by available online servers such as NetPhos (Blom et al., 1999) and NetPhosK (Blom et al., 2004). Interestingly, according with the number of the predicted serine, threonine, and tyrosine phosphorylation sites, PILS proteins can be grouped into three classes: (i) less than 10 (PILS5 and PILS7), (ii) between 10 and 15 (PILS2 and PILS6), and (iii) more than 15 (PILS1, PILS3, and PILS4). This finding may indicate the functional diversification among the PILS members and may suggest that different phosphorylation-based mechanisms are required for the regulation of PILS activity.

## Discussion

Auxin has pronounced importance for the plant development. Recent research shed light on a particular link between intracellular auxin transport processes and auxin metabolism (Mravec et al., 2009; Barbez et al., 2012; Bosco et al., 2012; Ding et al., 2012). Here, we report *in silico* analyses of PILS putative auxin flux facilitator sequences from *A. thaliana* and revealed certain features that might be functionally important for PILS activity.

The phylogenetic analysis of PILS sequences revealed that four *Physcomitrella* PILSes are found in Clade II, while only one is found in Clade III (Figure 2). Moreover, two *Selaginella* PILSes are found in each, Clade III and Clade II-PILS2 subclade, while four paralogs are found in the Clade II-PILS6 subclade (Figure 2). This, together with the distribution of the *Brachypodium, Oryza*, and *Arabidopsis* PILS sequences, indicates that the initial PILS divergence occurred in two separate clades already at the level of *Bryophytes*. We do not know if PILSes are present in the genome of Rhodophytes, but we can speculate that Clade II- and Clade III-PILSes may have originated before land plant evolution at the level of Streptophytes, as these algae are direct ancestors of land plants. Moreover, Clade II presumably diverged before or during the origin of Embryophytes, because this clade is already diversified in PILS2- and PILS6-like subclades in mosses (Figure 2). Clade III particularly expanded during higher plant evolution (Figure 2; Figure S1 in Supplementary Material). This clade is divided in PILS1/PILS3/PILS4 and PILS5/PILS7 subclades (Figure 2; Figure S1 in Supplementary Material). We could not estimate when these subclades emerged because PILS sequences from conifers and ferns are either incomplete (only ESTs available) or not available. More than 30000 ESTs derived from gametophyte of fern *Adiantum* can be found on NCBI but we could not identify any PILS sequence which indicates that PILSes might be not transcribed in gametophyte.

Combining the gene and protein analyses, *AtPILS4* is likely to be a recent duplication of *AtPILS3*, because they show very high amino acid identity (Table 2), strong gene collinearity (Figure 5A), and no particular *PILS4* orthologs could be identified in the genomes of the other sequenced species. *PILS3/PILS4* seem to be originally derived from *PILS1* (69% amino acid identity; Table 2). Accordingly, by analyzing the amino acid sequence similarities and the PILS phylogeny, we can conclude that from seven *PILSes* in *A. thaliana* genome, six in *Oryza sativa* ssp *japonica* and eight in *Brachypodium distachyon* genome only four are true orthologs. The other *PILS* members presumably represent lineage-specific duplications that occurred after the separation of the dicots and monocots about 200–250 *Mya*.

The existence of *PILS2* as a single-exon gene in most species is intriguing since single-exon genes are rather typical for prokaryotes. However, single-exon or intronless genes are present in eukaryotic genomes (Sakharkar et al., 2004) and can have many origins, but could pinpoint the relatedness to a prokaryotic gene (Zou et al., 2011). However, moss PILS2 orthologs display intronexon structure (Figure 7) and might suggest that *PILS2* genes lost the intron structure during evolution.

Our findings might highlight certain functional diversifications among PILS proteins. Notably, PILS2 and PILS5 have only 29% amino acid sequence identities (Table 2), display very diverged gene organization (Figure 6), and belong to diverse evolutionary sub clades (Figure 2). However, their gene regulation and function seem to be highly similar in *Arabidopsis*, because *PILS2* and *PILS5* have overlapping expression pattern in the root transition zone and redundantly control seedling growth and development (Barbez et al., 2012). Therefore, defined research is needed to evaluate the functional importance of the distinct features of the respective *PILS* genes and PILS proteins.

## Conflict of Interest Statement

The authors declare that the research was conducted in the absence of any commercial or financial relationships that could be construed as a potential conflict of interest.

## Supplementary Material

The Supplementary Material for this article can be found online at http://www.frontiersin.org/Plant_Traffic_and_Transport/10.3389/fpls.2012.00227/abstract

Supplementary Figure S1 **Molecular phylogenetic analysis of PILS proteins**. The diagram shows an extended phylogentic tree of PILS proteins with collapsed branches for algae, *Physcomitrella*, and *Selaginella*. Note the high diversification of PILSes in *Medicago* and *Populus*. Because of incomplete sequences some of the PILSes were eliminated. The evolutionary history was inferred by using the Maximum Likelihood method based on the Data specific model (Nei and Kumar, 2000). The tree with the highest log likelihood (−55875.7936) is shown. The percentage of trees in which the associated taxa clustered together is shown above the branches. Initial tree(s) for the heuristic search were obtained automatically as follows. When the number of common sites was <100 or less than one fourth of the total number of sites, the maximum parsimony method was used; otherwise BIONJ method with MCL distance matrix was used. A discrete Gamma distribution was used to model evolutionary rate differences among sites [five categories (+*G*, parameter = 2.6899)]. The rate variation model allowed for some sites to be evolutionarily invariable ([+I], 3.7299% sites). The tree is drawn to scale, with branch lengths measured in the number of substitutions per site. The analysis involved 75 nucleotide sequences. All positions with less than 0% site coverage were eliminated. That is, fewer than 100% alignment gaps, missing data, and ambiguous bases were allowed at any position. There were a total of 1113 positions in the final dataset. Evolutionary analyses were conducted in MEGA5 (Tamura et al., 2011).

Supplementary Figure S2 **Alignment of PILS amino acid sequences**. The multiple amino acid alignment of PILSes was generated by using Muscle in MEGA5 software (Tamura et al., 2011). This alignment was generated for the phylogenetic analysis presented in the Supplementary Figure 1. The alignment for the smaller tree presented in the Figure 2 is similar but with less sequences.

Supplementary Figure S3 **Alignment of PILS and PIN amino acid sequences**. The multiple alignment was generated by using Muscle in MEGA5 software (Tamura et al., 2011).

Supplementary Figure S4 **Alignment of *Arabidopsis thaliana* PILS amino acid sequences**. A multiple sequence alignment generated by ClustalW server (Larkin et al., 2007) of the seven PILSes is shown. Amino acids are color coded: red (small, hydrophobic, aromatic, not Y), blue (acidic); magenta (basic), green (hydroxyl, amine, amide, basic), gray (others). “*,” Identical amino acids; “:,” conserved substitutions (same color group); “.,” semi-conserved substitution (similar shapes).

Supplementary Table S1 **Sequence information**.

## Footnotes

^1^http://bioinformatics.psb.ugent.be/plaza/

^2^http://www.ncbi.nlm.nih.gov/

^3^http://www.phytozome.net/

^4^http://www.r-project.org/

^5^www.genevestigator.com

^6^http://www.arabidopsis.org
