# Supplementary material for: Evolution and Structural Diversification of PILS Putative Auxin Carriers in Plants
Source: Front Plant Sci. 2012 Oct 12;3:227. doi: 10.3389/fpls.2012.00227 (PMC3470039; doi:10.3389/fpls.2012.00227)
Supplement: Supplementary Figure S1 — Molecular phylogenetic analysis of PILS proteins. The diagram shows an extended phylogentic tree of PILS proteins with collapsed branches for algae, Physcomitrella, and Selaginella. Note the high diversification of PILSes in Medicago and Populus. Because of incomplete sequences some of the PILSes were eliminated. The evolutionary history was inferred by using the Maximum Likelihood method based on the Data specific model (Nei and Kumar, 2000). The tree with the highest log likelihood (−55875.7936) is shown. The percentage of trees in which the associated taxa clustered together is shown above the branches. Initial tree(s) for the heuristic search were obtained automatically as follows. When the number of common sites was <100 or less than one fourth of the total number of sites, the maximum parsimony method was used; otherwise BIONJ method with MCL distance matrix was used. A discrete Gamma distribution was used to model evolutionary rate differences among sites [five categories (+G, parameter = 2.6899)]. The rate variation model allowed for some sites to be evolutionarily invariable ([+I], 3.7299% sites). The tree is drawn to scale, with branch lengths measured in the number of substitutions per site. The analysis involved 75 nucleotide sequences. All positions with less than 0% site coverage were eliminated. That is, fewer than 100% alignment gaps, missing data, and ambiguous bases were allowed at any position. There were a total of 1113 positions in the final dataset. Evolutionary analyses were conducted in MEGA5 (Tamura et al., 2011). [file 32149_Kleine-Vehn_DataSheet1.PDF]

Supplementary Figure 1

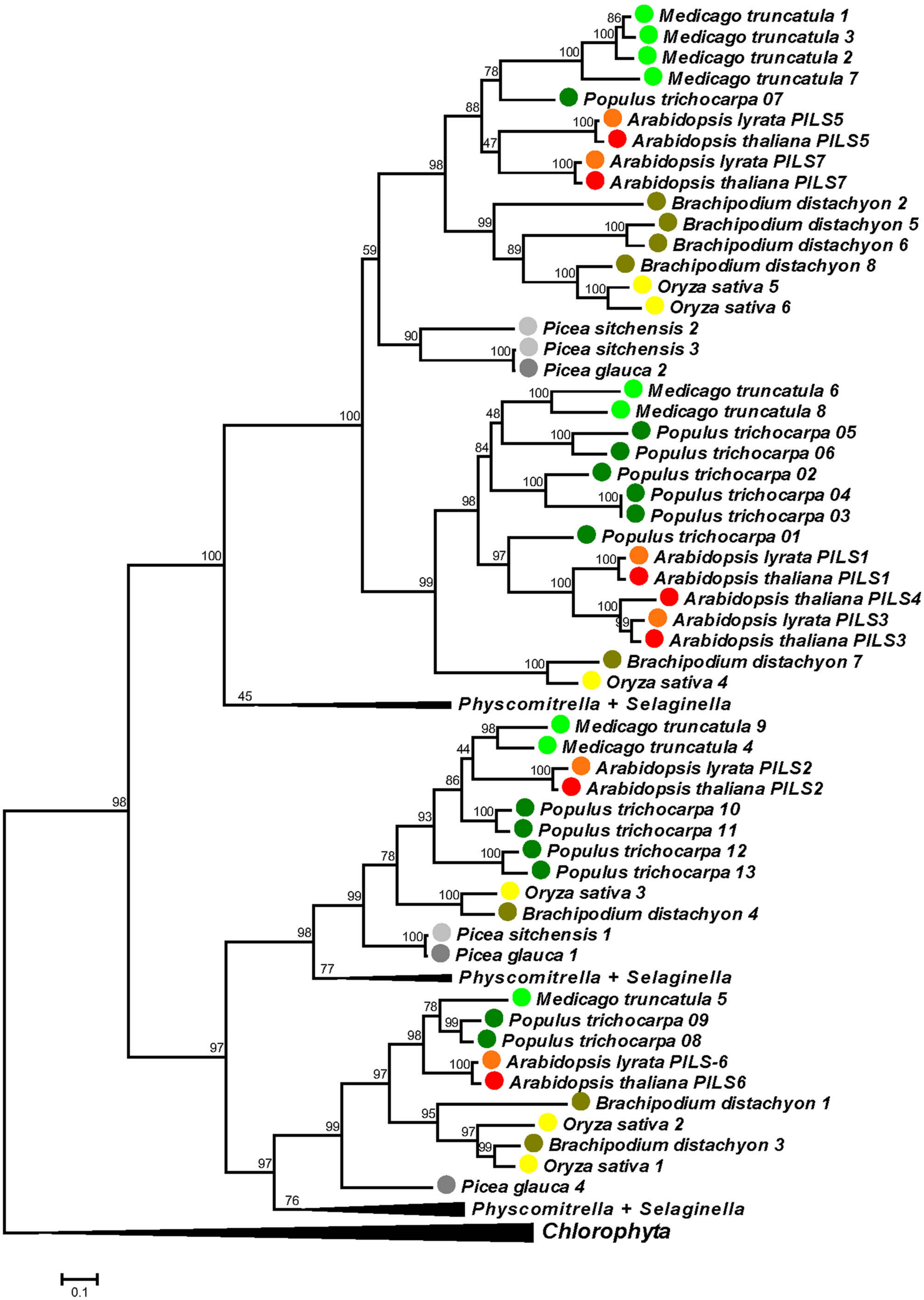

Supplementary Figure 2

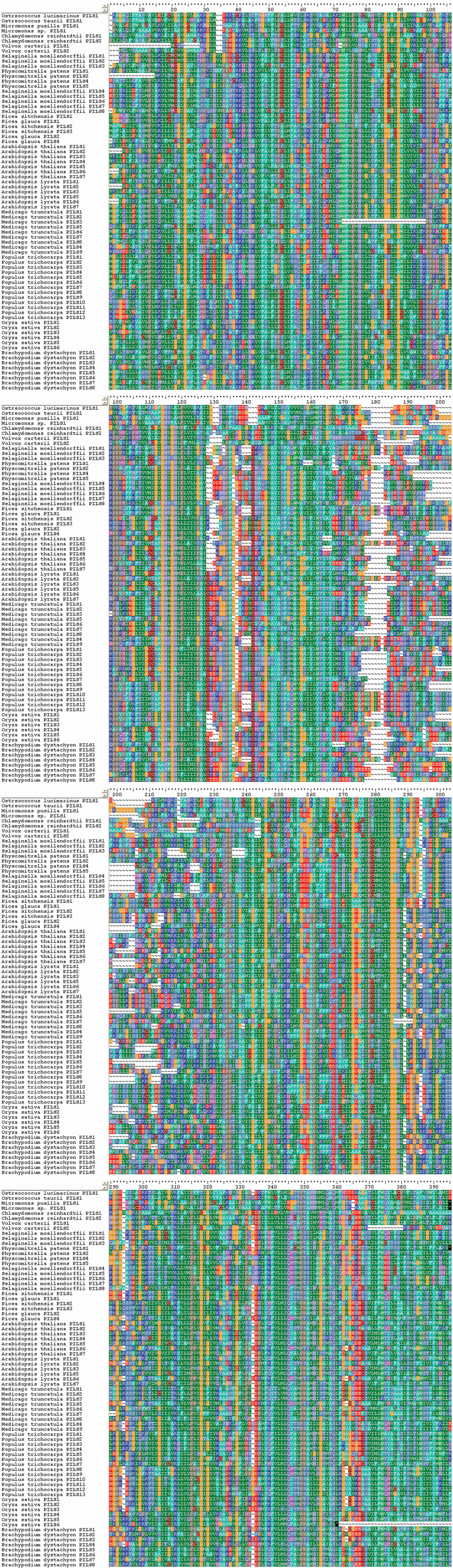

Supplementary Figure 3

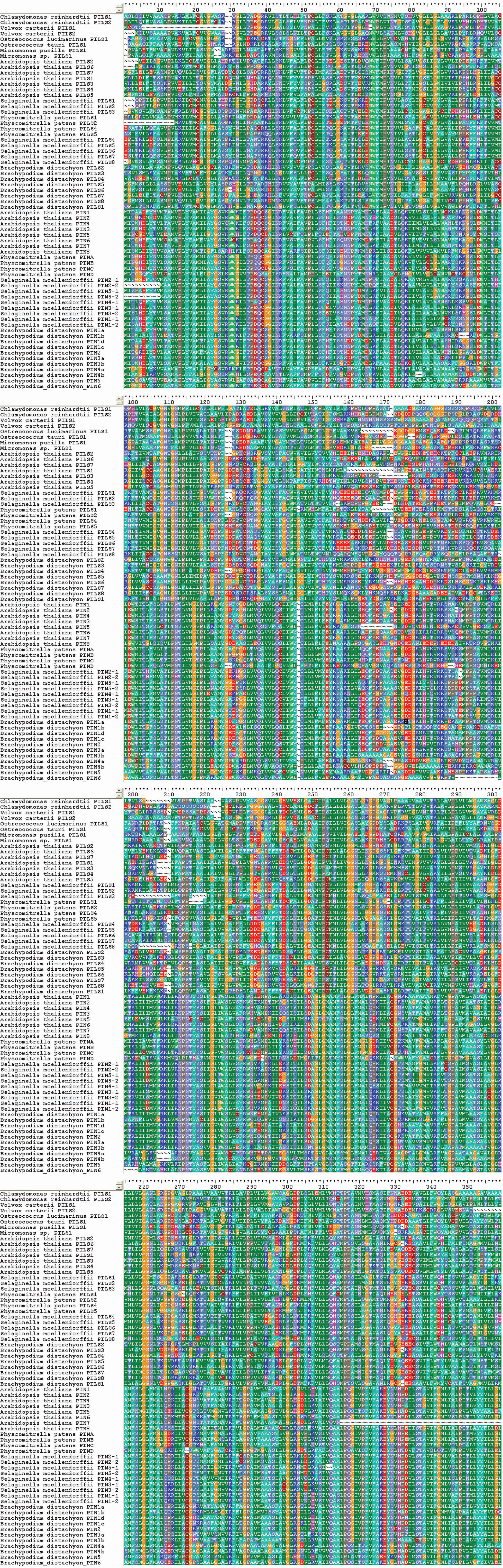

Supplementary Figure 4

```
Ath_PILS2       ------------------------------------------------------------
Ath_PILS6       ------------------------------------------------------------
Ath_PILS3       ------------------------------------------------------------
Ath_PILS4       ------------------------------------------------------------
Ath_PILS1       MSLTQSAKLHIIHQSIGKITTRLYDTSPFHGDSHCKQRRVKDNCIQFNMNISNVLKCYSE 60
Ath_PILS5       ------------------------------------------------------------
Ath_PILS7       ------------------------------------------------------------


Ath_PILS2       --------------------MSGFSSGNVNSRVVDILSG-----------VVPLLKLICL 29
Ath_PILS6       -------------------MIARILAALADSMEMPVAAGGGSVLGTIKIAVMPIAKVFTM 41
Ath_PILS3       ---------------------------MVKLLELFITSS------------KPVVEILLI 21
Ath_PILS4       ----------------------------MKLLELFIASS------------KPVVETLLI 20
Ath_PILS1       IESFSINQRIRRSISLFLSILRSFYSMRMRLLDLFITSS------------IPVAKILLI 108
Ath_PILS5       ----------------------------MGFWSLLEVAS------------MPVIQVLFM 20
Ath_PILS7       ----------------------------MGFLELLEVAS------------MPIVQVLLI 20
                                                 :   :.             *: : : :

Ath_PILS2       TVIGLLLAHPKTQLVPRATFRLLSKLVFALFLPCLIFTELGESITLDNIVQWWFIPVNVL 89
Ath_PILS6       CFLGLLMASKYVNILPPSGRKLLNGLVFSLLLPCLIFSQLGQAVTLQKMLQWWFIPVNVV 101
Ath_PILS3       TSVGFYMALDGVNLLGHDARKYLNNIVFYVFSPSLIGSRLADSVTYESLVKMWFMPVNVL 81
Ath_PILS4       TSVGFYLALDTVNLLGHDARKHLNNIVFYVFSPSLIGSRLADSVTYESLVKMWFMPVNVL 80
Ath_PILS1       TGIGFYLALDQVNILNHDARKQLNNIVFYVFSPSLVASSLSETITYESMVKMWFMPLNVL 168
Ath_PILS5       SLVGAFMASDRCKLFPVEARNSMNKVVFVLFAPALMFANLAQTVTLEDIISWWFMPVNMG 80
Ath_PILS7       SVLGAFLATDYCSLLSADTRRSVNKLVFVVFTPCIMFANLAETVTLQDIISWWFMPINVG 80
                  :*  :*    .:.     . :. :** :: *.:: : *.:::* :.::. **:*:*:

Ath_PILS2       LSAVVGSLIGYLVVLICRPPPEFNRFTIVMTAFGNTGNLLLAIVSSVCHTKTNPFG--PN 147
Ath_PILS6       LGTISGSIIGFIVASIVRPPYPYFKFTIIQIGVGNIGNVPLVLLAALCRDTSNPFGDSEK 161
Ath_PILS3       LTFIIGSLLGWIVIVITKPPSHLRGLILGCCAAGNLGNMPLIIIPAVCKEKGGPFGDPES 141
Ath_PILS4       LTFMIGSLLGWIVIVITKPPSQLRGLIISCCASGNLGTMPLIIIPAICKEKGGPFGDSES 140
Ath_PILS1       LTFIIGSFLGWIVIKITKPPSHLRGIIVGCCAAGNLGNMPLIIIPAICNEKGSPFGDPES 228
Ath_PILS5       LTFLIGGLLGWLVVKILKPPPYLEGLIVATCSAGNMGNLPIILVPAICDEDKSPFGNRSV 140
Ath_PILS7       ITFLVGGILGWLVVKLLNPKPQLHGLIIATCASGNMGNLMLILVPAICDEEGSPFGNRSV 140
                :  : *.::*::*  : .*      : :   . ** *.: : ::.::*    .***

Ath_PILS2       CNSRGVSYVSFAQWVAVILVYT-----VVYHMMEP-------PLEYYEVVEEEGVEIEEI 195
Ath_PILS6       CSIDGTAYISFGQWVGAIILYT-----YVYQMFAP-------PPEGFDAEEEN-LALKTL 208
Ath_PILS3       CQKYGMGYVALSMAMGSIYIWT-----YVY-----------NLMRVLS-NSPVETPP-SV 183
Ath_PILS4       CEKYGMGYVTLSMTAFFISVYKHDTNWYVSGGNGLLMDLYINLMRVLS-NSPVETHTHSI 199
Ath_PILS1       CEKFGLGYIALSMAIGAIYIWT-----YVY-----------NLMRMLA-NPAGETAINST 271
Ath_PILS5       CRTVGLSYASFSMALGGFYIWT-----YTFRLIKG------SAMKVQAIEESEKIAIKSS 189
Ath_PILS7       CRSIGLSYASFSMALGGFYIWT-----YSYQLVRS------SATQFRALEAAG--LVKSP 187
                *   * .* ::.     : ::.                      .

Ath_PILS2       NVENHDASRPLLVEAEWPGIEDKETEHCKTPFIARVFNSISSFSQTSFPEVDLGGEYGGE 255
Ath_PILS6       PVDAAPEQVPLLTQN---------------------------FPKDFSPTQDL---LPVQ 238
Ath_PILS3       ESNYD-SYKVPLISSKEEE----------------------------------------- 201
Ath_PILS4       ESNYDDSCKVQLISSKEEEK---------------------------------------- 219
Ath_PILS1       SSTMP------LISPKVEV----------------------------------------- 284
Ath_PILS5       NSDLEADHKTHLLGAPEDK----------------------------------------- 208
Ath_PILS7       NKDIDSDPHALLLKPHQNQ----------------------------------------- 206
                           *

Ath_PILS2       SSSPRSIQCLAEPRVMRRIRVVAEQTPVKHILQPPTIASLLAIIIGSVPQLKSVVFGYDA 315
Ath_PILS6       STEPRGRGVSRKGKIAQIFVFLYEKLKLKQIVQPAIVASILAMILGAIPFTKKLIFTNGA 298
Ath_PILS3       ----NNQKAGRWEKVKRRLVSLSQKVNLKTIFAPSTIAAMIALVIGLITPLRKLIIGTEA 257
Ath_PILS4       --EEDNHQVGRWEEVKQRVVSLSKKVNLGSIFAPATIAAIIALVIGLITPLRNLIIGTVA 277
Ath_PILS1       -----AEQVGTWGKVKQRVCSVAEKINLRTIFAPSTIAALIALAVGLNPLLRKLLVGNTA 339
Ath_PILS5       ----ENKVV-KEKTGFWRKGVDFLHEILEELLAPPTLGAIIGFIFGAVRWLRNLIIGDDA 263
Ath_PILS7       ----DLEIQGKQKVSTRTYIKDLLHQILEELFAPPTIGAILGFVFGATNWLRNLIIGENA 262
                                        :  :  :. *. :.:::.: .*     :.::.   *
Ath_PILS2       PLSFITDSLNIMGSAMVPSVMLVLGGMLSEGPN-----ESTLGLRTTIGISVARLLVLPL 370
Ath_PILS6       PLFFFTDSCMILGDAMIPCILLALGGNLINGPG-----SSKLGFKTTAAIIIGRLVLVPP 353
Ath_PILS3       PLRVLQDSVTLVGDGAVPAMTMIIGGNLLKGLR-----SSGMKMSSIIGVLVARYVLLPM 312
Ath_PILS4       PFRVIQDSLTLLGDGAIPAMTLILGGNLLKGMRRSEVRSSEMKNSCIIGVLVARYILLPV 337
Ath_PILS1       PLRVIEDSVSLLGDGAIPVLTLIVGGNLLNGLR-----GSGINKSVIMGVVVVRYLLLPI 394
Ath_PILS5       PLRIVQSTAKLLGDGTIPCMTIILGGNLIQGLR-----SSAVKPMVVLGIVCVRYIAMPI 318
Ath_PILS7       PLRVIQDSVKLLGEGTIPCITLILGGNLIQGLR-----SSAVKKSVIVGVIIVRYILLPV 317
                *: .. .:  ::*.. :* : : :** * :*        * :      .:   * : :*

Ath_PILS2       VGIGIVMSADKLGLISSADPMFKFVLLLQYSTPSAILLGAIASLRGYAVREASALLFWQH 430
Ath_PILS6       VGLGIVTVADKLGFLPADDKMFRFVLLLQHTMPTSVLSGAVANLRG-CGRESAAVLFWVH 412
Ath_PILS3       SGVLIVRGAYKLDLVT-SEPLYQFVLLLQYAVPPAMNLGTITQLFGTGESECSVIMLWTY 371
Ath_PILS4       SGVLLVRGAYKLDLVT-SEPLYQFVLLLQYAVPPAMNLGTKTQLFGAGESECSVIMLWTY 396
Ath_PILS1       LGVFIVRGAHYLGLVT-SEPLYQFVLLLQYVVPPAMNLGTITQLFGSGESECSVILFWSY 453
Ath_PILS5       IGIGIVLTAANLGFLP-ADPLFQYVLMLQFTLPPAMNIGTMTQLYNVAQDECSVLMLWTY 377
Ath_PILS7       VGVGVVQLAGNLGYLP-PDPLFRYVLMLQFALPPAMNISTMAQLFDVAQDECSVIFLWTY 376
                 *: :*  *  *. :.  : ::::**:**.  *.::  .: :.* .    *.:.:::* :

Ath_PILS2       IFALLSLTFYIVIFFKLTVETTVQGMQ 457
Ath_PILS6       IFAIFSMAGWMVLYINILF-------- 431
Ath_PILS3       SLASIALTVWPTFFMWLVA-------- 390
Ath_PILS4       SLAAVSLTVWPTFFMWLVT-------- 415
Ath_PILS1       ALASVSLTVWPTFFMWLVA-------- 472
Ath_PILS5       LVAILALTVWSTIFLHLLV-------- 396
Ath_PILS7       LVASLALTVWSTIFLSILS-------- 395
                 .* .::: : .::: :
```

Supplementary Table 1

Sequence information

Taxon	PILS/PIN number	DatabaseID (NCBI, PLAZA or Phytozome)
Arabidopsis lyrata	PILS1	gi|297845069|ref|XM_002890370.1|
Arabidopsis lyrata	PILS2	gi|297838924|ref|XM_002887298.1|
Arabidopsis lyrata	PILS3	gi|297839524|ref|XM_002887598.1|
Arabidopsis lyrata	PILS5	gi|297832359|ref|XM_002884016.1|
Arabidopsis lyrata	PILS6	gi|297806008|ref|XM_002870842.1|
Arabidopsis lyrata	PILS7	gi|297797790|ref|XM_002866734.1|
Arabidopsis thaliana	PILS1	AT1G20925
Arabidopsis thaliana	PILS2	AT1G71090
Arabidopsis thaliana	PILS3	AT1G76520
Arabidopsis thaliana	PILS4	AT1G76530
Arabidopsis thaliana	PILS5	AT2G17500
Arabidopsis thaliana	PILS6	AT5G01990
Arabidopsis thaliana	PILS7	AT5G65980
Brachypodium distachyon	PILS1	Bdi1G63446
Brachypodium distachyon	PILS2	gi|357120862|ref|XM_003562096.1|
Brachypodium distachyon	PILS3	gi|357125608|ref|XM_003564436.1|
Brachypodium distachyon	PILS4	gi|357126973|ref|XM_003565114.1|
Brachypodium distachyon	PILS5	gi|357138294|ref|XM_003570683.1|
Brachypodium distachyon	PILS6	gi|357144102|ref|XM_003573124.1|
Brachypodium distachyon	PILS7	gi|357159092|ref|XM_003578288.1|
Brachypodium distachyon	PILS8	gi|357159956|ref|XM_003578564.1|
Chlamydomonas reinhardtii	PILS1	Cre13G00630
Chlamydomonas reinhardtii	PILS2	gi|159475550|ref|XM_001695830.1|
Medicago truncatula	PILS1	gi|357484232|ref|XM_003612356.1|
Medicago truncatula	PILS2	gi|357484228|ref|XM_003612354.1|
Medicago truncatula	PILS3	gi|357484206|ref|XM_003612343.1|
Medicago truncatula	PILS4	gi|357488456|ref|XM_003614468.1|
Medicago truncatula	PILS5	gi|357506748|ref|XM_003623615.1|
Medicago truncatula	PILS6	gi|357484286|ref|XM_003612383.1|
Medicago truncatula	PILS7	gi|357517624|ref|XM_003629053.1|
Medicago truncatula	PILS8	gi|357517518|ref|XM_003629000.1|
Medicago truncatula	PILS9	gi|357444542|ref|XM_003592501.1|
Micromonas pusilla	PILS1	gi|303276958|ref|XM_003057727.1|
Micromonas sp.	PILS1	gi|255072982|ref|XM_002500120.1|
Oryza sativa	PILS1	gi|115440694|ref|NM_001051162.1|
Oryza sativa	PILS2	gi|115464530|ref|NM_001062400.1|
Oryza sativa	PILS3	gi|115475144|ref|NM_001067704.1|
Oryza sativa	PILS4	gi|297727104|ref|NM_001188987.1|
Oryza sativa	PILS5	gi|115480586|ref|NM_001070422.1|
Oryza sativa	PILS6	gi|297609931|ref|NM_001070423.2|
Ostreococcus lucimarinus	PILS1	gi|145348613|ref|XM_001418704.1|
Ostreococcus taurii	PILS1	gi|308806212|ref|XM_003080370.1|
Physcomitrella patens	PILS1	gi|168028602|ref|XM_001766765.1|
Physcomitrella patens	PILS2	gi|168043601|ref|XM_001774221.1|
Physcomitrella patens	PILS3	Ppa00225G00240
Physcomitrella patens	PILS4	gi|168065598|ref|XM_001784685.1|
Physcomitrella patens	PILS5	gi|168067512|ref|XM_001785607.1|
Picea glauca	PILS1	Contig-0 from EST
Picea glauca	PILS2	Contig-1 from EST
Picea glauca	PILS4	Contig-3 from EST
Picea sitchensis	PILS1	gi|224286947|gb|BT071727.1|
Picea sitchensis	PILS2	gi|148906465|gb|EF676485.1|
Picea sitchensis	PILS3	gi|148910205|gb|EF678427.1|
Populus trichocarpa	PILS1	gi|224065632|ref|XM_002301858.1|
Populus trichocarpa	PILS2	gi|224061558|ref|XM_002300504.1|
Populus trichocarpa	PILS3	gi|224115415|ref|XM_002316993.1|
Populus trichocarpa	PILS4	gi|118481906|gb|EF144662.1|
Populus trichocarpa	PILS5	gi|224071516|ref|XM_002303462.1|
Populus trichocarpa	PILS6	gi|224117651|ref|XM_002317598.1|
Populus trichocarpa	PILS7	gi|224081466|ref|XM_002306385.1|
Populus trichocarpa	PILS8	gi|224140640|ref|XM_002323654.1|
Populus trichocarpa	PILS9	gi|224069081|ref|XM_002326234.1|
Populus trichocarpa	PILS10	gi|224101808|ref|XM_002312393.1|
Populus trichocarpa	PILS11	gi|224108350|ref|XM_002314781.1|
Populus trichocarpa	PILS12	gi|224065211|ref|XM_002301683.1|
Populus trichocarpa	PILS13	gi|224079396|ref|XM_002305815.1|
Selaginella moellendorffii	PILS1	gi|302763116|ref|XM_002964934.1|
Selaginella moellendorffii	PILS2	gi|302790573|ref|XM_002977008.1|
Selaginella moellendorffii	PILS3	gi|302800142|ref|XM_002981783.1|
Selaginella moellendorffii	PILS4	gi|302753651|ref|XM_002960204.1|
Selaginella moellendorffii	PILS5	gi|302768089|ref|XM_002967419.1|
Selaginella moellendorffii	PILS6	gi|302807693|ref|XM_002985495.1|
Selaginella moellendorffii	PILS7	gi|302810674|ref|XM_002986982.1|
Selaginella moellendorffii	PILS8	gi|302802084|ref|XM_002982752.1|
Volvox carterii	PILS1	gi|302835793|ref|XM_002949412.1|
Volvox carterii	PILS2	gi|302852558|ref|XM_002957753.1|
Arabidopsis thaliana	PIN1	gi|145337498|ref|NM_106017.3|
Arabidopsis thaliana	PIN2	gi|145359343|ref|NM_125091.3|
Arabidopsis thaliana	PIN3	gi|30698766|ref|NM_105762.2|
Arabidopsis thaliana	PIN4	gi|30677919|ref|NM_179592.1|
Arabidopsis thaliana	PIN5	gi|240256297|ref|NM_121659.4|
Arabidopsis thaliana	PIN6	gi|186495961|ref|NM_106361.3|
Arabidopsis thaliana	PIN7	gi|18395199|ref|NM_102156.1|
Arabidopsis thaliana	PIN8	gi|18417566|ref|NM_121514.1|
Physcomitrella patens	PINA	gi|168001819|ref|XM_001753560.1|
Physcomitrella patens	PINB	gi|168005647|ref|XM_001755470.1|
Physcomitrella patens	PINC	gi|168011235|ref|XM_001758257.1|
Physcomitrella patens	PIND	gi|168026486|ref|XM_001765711.1|
Selaginella moellendorffii	PIN1-1	gi|302804002|ref|XM_002983708.1|
Selaginella moellendorffii	PIN1-2	gi|302817651|ref|XM_002990455.1|
Selaginella moellendorffii	PIN2-1	gi|302786531|ref|XM_002974991.1|
Selaginella moellendorffii	PIN2-2	gi|302791380|ref|XM_002977411.1|
Selaginella moellendorffii	PIN3-1	gi|302783123|ref|XM_002973289.1|
Selaginella moellendorffii	PIN3-2	gi|302789867|ref|XM_002976656.1|
Selaginella moellendorffii	PIN4-1	gi|302804076|ref|XM_002983745.1|
Selaginella moellendorffii	PIN5-1	gi|302768324|ref|XM_002967536.1|
Selaginella moellendorffii	PIN5-2	gi|302800009|ref|XM_002981717.1|
Brachypodium distachyon	PIN1a	gi|357138160|ref|XM_003570618.1|
Brachypodium distachyon	PIN1b	gi|357136172|ref|XM_003569632.1|
Brachypodium distachyon	PIN1c	gi|357124704|ref|XM_003563990.1|
Brachypodium distachyon	PIN1d	gi|357157667|ref|XM_003577827.1|
Brachypodium distachyon	PIN2	gi|357123278|ref|XM_003563291.1|
Brachypodium distachyon	PIN3a	gi|357135686|ref|XM_003569392.1|
Brachypodium distachyon	PIN3b	gi|357128413|ref|XM_003565820.1|
Brachypodium distachyon	PIN4a	gi|357154215|ref|XM_003576662.1|
Brachypodium distachyon	PIN4b	gi|357148559|ref|XM_003574765.1|
Brachypodium distachyon	PIN5	gi|357125469|ref|XM_003564369.1|
Brachypodium distachyon	PIN6	gi|357126529|ref|XM_003564892.1|
